# Copolymer of Phenylene and Thiophene toward a Visible‐Light‐Driven Photocatalytic Oxygen Reduction to Hydrogen Peroxide

**DOI:** 10.1002/advs.202003077

**Published:** 2021-01-20

**Authors:** Kouki Oka, Hiroyuki Nishide, Bjorn Winther‐Jensen

**Affiliations:** ^1^ Department of Applied Chemistry and Research Institute for Science and Engineering Waseda University 3‐4‐1 Okubo Shinjuku Tokyo 169‐8555 Japan

**Keywords:** oxygen reduction, polythiophenes, *π*‐conjugated polymers

## Abstract

*π*‐Conjugated polymers including polythiophenes are emerging as promising electrode materials for (photo)electrochemical reactions, such as water reduction to H_2_ production and oxygen (O_2_) reduction to hydrogen peroxide (H_2_O_2_) production. In the current work, a copolymer of phenylene and thiophene is designed, where the phenylene ring lowers the highest occupied molecular orbital level of the polymer of visible‐light‐harvesting thiophene entities and works as a robust catalytic site for the O_2_ reduction to H_2_O_2_ production. The very high onset potential of the copolymer for O_2_ reduction (+1.53 V vs RHE, pH 12) allows a H_2_O_2_ production setup with a traditional water‐oxidation catalyst, manganese oxide (MnO*_x_*), as the anode. MnO*_x_* is deposited on one face of a conducting plate, and visible‐light illumination of the copolymer layer formed on the other face aids steady O_2_ reduction to H_2_O_2_ with no bias assistance and a complete photocatalytic conversion rate of 14 000 mg (H_2_O_2_) g_photocat_^−1^ h^−1^ or ≈0.2 mg (H_2_O_2_) cm^−2^ h^−1^.

## Graphical abstract

Metal‐free copolymer of phenylene and thiophene (PPT) works as a visible‐light‐harvester and highly selective and robust catalyst for the oxygen reduction to hydrogen peroxide (H_2_O_2_). Visible‐light‐illuminated PPT layer in combination with a manganese oxide (MnO*_x_*) catalyst exhibits the steady H_2_O_2_ production without bias voltage at pH 12 and with a very high gravimetric photocatalytic conversion rate of 14 000 mg (H_2_O_2_) g_photocat_^−1^.

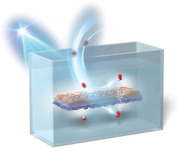

## 1 Introduction

H_2_O_2_ is widely used in pulp and paper bleaching, textile processing, production of electronics, and wastewater treatment, and as a metal‐free oxidant in chemical syntheses.^[^ ^1^ ^]^ The oxidizing power of hydrogen peroxide (H_2_O_2_) enables the decontamination of biological contaminates, including SARS‐CoV‐2, which causes the COVID‐19 disease.^[^ ^2^ ^]^ H_2_O_2_ is also a promising alternative to hydrogen (H_2_) as a cleaner, safer energy carrier.^[^ ^3^ ^]^ The annual global market for H_2_O_2_ is 3 million metric tons, which is expected to double by 2022.^[^ ^4^ ^]^ However, the use of H_2_O_2_ as a clean chemical reagent and/or energy carrier is feasible only if it can be produced in a sustainable and on‐site manner^[^ ^1^, ^5^ ^]^ and not via the current anthraquinone route, where high‐pressure H_2_ derived from methane (CH_4_) is used as feedstock and large amounts of aromatic solvents are required for the complicated H_2_O_2_ extraction process.^[^ ^1^, ^6^ ^]^

Much effort has been directed at solar‐driven photochemical H_2_O_2_ production from oxygen (O_2_) and water, a simple and green process with low energy consumption.^[^ ^7^ ^]^ Inorganic semiconductors for photochemical H_2_O_2_ production, such as ZnO,^[^ ^7a,b^ ^]^ have long been a topic of research interest, but they have insufficient photocatalytic abilities, thus requiring the use of sacrificial agents such as formate and phenol without exception. Recent research is aimed at overcoming these difficulties. For example, the polyoxometalate cluster [PW_11_O_39_]^7−^ was covalently combined with 3D ordered microporous graphitic carbon nitride via an organic linker to realize the highest catalytic rate of 12 mg (H_2_O_2_) g_photocat_^−1^ h^−1^ without any sacrificial agents^[^ ^7c^ ^]^ (the H_2_O_2_ production efficiency of different photocatalysts and catalytic systems is quantified in terms of the gravimetric rate, which is expressed in mg (H_2_O_2_) g_photocat_^−1^ h^−1^ and often determined in water under 1 atm O_2_). However, the performance of solar‐driven photochemical H_2_O_2_ production remains low (<15 mg (H_2_O_2_) g_photocat_^−1^ h^−1^).

Photo‐electrocatalysts or photocathodes for the reduction of O_2_ to H_2_O_2_ have also been investigated to achieve higher catalytic rates under illumination in the presence of an additional bias potential. ^[^ ^8^ ^]^ The typical examples in this regard are organic epindolidione pigments^[^ ^8a^ ^]^ and dye‐sensitized nickel oxides^[^ ^8b^ ^]^. Although the production rates exceed those of previous photocatalysts^[^ ^7c‐g^ ^]^ by one to two orders of magnitude, the corresponding devices require a significant bias voltage and must be operated at pH ≤ 7 because of the instability of the catalysts under alkaline conditions. A practical electrochemical device will require a counter electrode for the anode reaction of oxygen reduction, preferably catalytic oxygen evolution in water. As traditional water oxidation catalysts such as manganese oxide (MnO*_x_*)^[^ ^9^ ^]^ and CoO*_x_* ^[^ ^10^ ^]^ function optimally at high pH, a photocatalytic cathode which can function at high pH and yield a photovoltage exceeding the voltage between O_2_ reduction to H_2_O_2_ and water oxidation would be ideal.

*π*‐Conjugated polymers are emerging as appealing (photo)electrocatalytic materials.^[^ ^11^ ^]^ For example, we succeeded in forming polythiophene layers as a photo(electro)catalyst with an appropriate band structure and visible‐light absorption ability for the reduction of water to H_2_ at high pH,^[^ ^11d‐g^ ^]^ which relies on light to initiate the catalytic reaction. Polythiophenes have also been studied as an organic electrocatalyst for O_2_ reduction to H_2_O_2_ in the dark.^[^ ^12^ ^]^ Experimental and simulation results suggest the thiophene units as a catalytic site. Very recently, a record‐high H_2_O_2_ concentration of 110 × 10^−3^ m was achieved with the photo‐electrocatalytic O_2_ reduction on polyterthiophene after the sustained reaction for 11 h at high pH.^[^ ^13^ ^]^ A dual‐photoelectrode device was also developed by using polythiophene, the water‐oxidation photocatalyst for NiFeO*_x_*, BiVO_4_, and a Nafion membrane.

**Figure 1:**
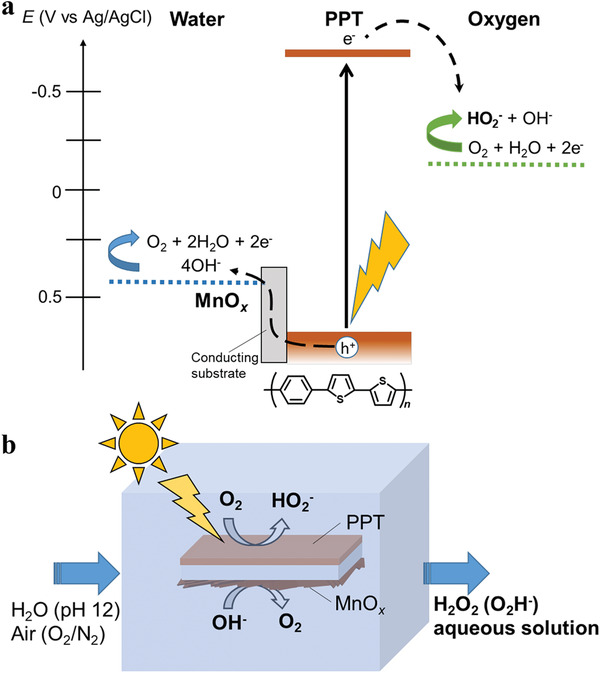
Combination of PPT photocathode and MnO*_x_* anode on a single plate. a) Energy diagram of the system. b) Photocatalytic H_2_O_2_ production at pH 12 on a single transparent, electrically conducting thin plate sandwiched between a PPT polymer layer for O_2_ reduction under illumination and MnO*_x_* for water oxidation.

However, it is challenging to accomplish photocatalytic O_2_ reduction to H_2_O_2_, which involves the use of a single photocatalytic *π*‐conjugated polymer possessing sufficient photovoltage to trigger the reduction without any bias voltage, another photocatalyst, and any suitable device configuration. One way to improve the photovoltage of the catalyst polymer is by lowering the highest occupied molecular orbital (HOMO) level of the polymer. We recently succeeded in the molecular design for lowering the HOMO level of visible‐light harvesting polythiophenes by copolymerization with an electron‐withdrawing phenylene unit, poly[1,4‐phenylene‐*alt*‐(2,2′‐bisthiophene)‐5,5′‐diyl] (PPT).^[^ ^11d^ ^]^ Herein, we demonstrate our successful use of the purely organic PPT polymer layer as a complete photocatalyst under visible‐light for O_2_ reduction to steady H_2_O_2_ production with high selectivity. The very high onset potential for the O_2_ reduction allows a facile setup with the traditional water‐oxidation counterpart catalyst, MnO*_x_*, as the anode (**Figure** **1**a). Sustained H_2_O_2_ production is enabled for the PPT photocatalyst polymer layer coated on the electrically conductive thin plate with the deposition of MnO*_x_* on its dark backside face and by simply soaking the plate in alkaline water under visible light irradiation (Figure 1b).

## 2 Results and Discussion

### 2.1 Photo‐electrocatalytic Performance of PPT for H_2_O_2_ Production

The PPT polymer layers were prepared by the polymerization of 1,4‐di(2‐thienyl)benzene spin‐coated on a glassy carbon or an ITO glass plate with iodine vapor as oxidant to avoid contamination by metals. The samples were carefully washed to remove residual iodine and monomer, yielding undoped and genuine PPT layers with thicknesses of 10–210 nm (for details on preparation and characterization, see the Supplementary Methods section in the Supporting Information). The PPT layers were deep brown in color, with a visible absorption edge at ≈570 nm (Figure S1, Supporting Information). The HOMO and LUMO levels were estimated to be −5.5 and −3.3 eV and −5.2 and −3.2 eV for PPT and polyterthiophene, respectively. The HOMO level was lowered with 0.3 V by introducing the electron‐withdrawing phenylene unit in the copolymer structure of the visible‐light‐harvesting thiophene polymer.

**Figure 2:**
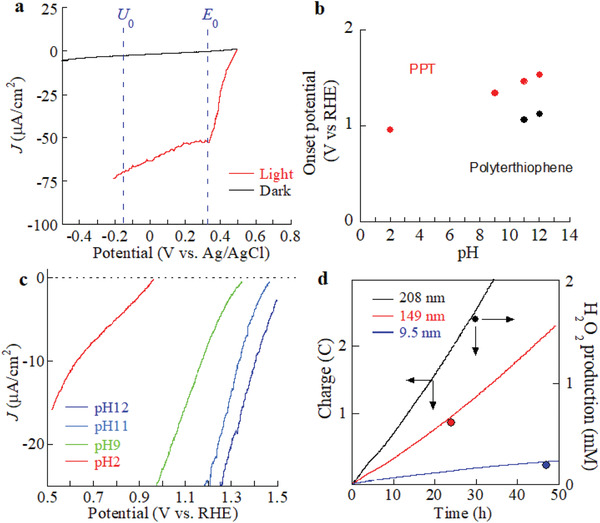
Photo‐electrocatalytic properties of PPT. a) Linear sweep voltammograms of the PPT photocathode recorded at 1 mV s^−1^ and pH 12 with and without light. *E* ^0^ refers to the thermodynamic water‐oxidation potential at pH 12. A low scan rate (1 mV s^−1^) was maintained to suppress capacitive effects, and the electrolyte ionic strength was maintained for all pH levels using NaCl as a supporting electrolyte. *J* is current density derived from the O_2_ reduction reaction. b) Open‐circuit potential of the PPT photocathode at different pH levels. c) Linear sweep voltammograms of the PPT photocathode recorded at 1 mV s^−1^ and different pHs. For the investigated pH range, the potential of the O_2_ reduction reaction under illumination increased by 0.9–1.0 V (measured at 10 µA cm^−2^) compared with the values observed in the dark. d) H_2_O_2_ production on various PPT layers (9.5, 149, and 208 nm thicknesses), estimated by chronoamperometry and titration (at 0 V vs Ag/AgCl and pH 12 using an electrochemical cell with a volume of 10 mL).

First, the photocatalytic performance of PPT on glassy carbon for H_2_O_2_ production was examined in the dark and under illumination at high pH with air bubbling (oxygen/nitrogen gas mixture). A traditional water‐oxidation catalyst worked as the counterpart in the full‐cell setup (linear sweep voltammetry results in **Figure** **2**a; see Figure S2 (Supporting Information) for the electrocatalytic performance in the dark). The O_2_ reduction was monitored from +0.6 V versus Ag/AgCl, which is beyond the thermodynamic water‐oxidation potential (+0.3 V vs Ag/AgCl represented with *E*° and the blue dashed line), at pH 12 under illumination (Figure 2a). Remarkably, the onset for light‐assisted O_2_ reduction of PPT (+1.53 V vs RHE at pH 12 in Figure 2b) was enhanced in comparison with that for polyterthiophene (+1.13 V vs RHE at pH 12), as expected from the lowered HOMO level, and significantly exceeded +1.23 V versus RHE for the thermodynamic water‐oxidation potential^[^ ^14^ ^]^ at pH 12, which is sufficient to drive water oxidation on the anode without any additional bias. A high pH was found to benefit the photo‐electrocatalytic activity of the PPT film (Figure 2c), which suggested that protons are not involved in the reaction, and that oxygen and water are the reactants, as described by Equation (1) and Equation (2).^[^ ^15^ ^]^ Here, *U* ^0^ is the standard equilibrium potential for each reaction, calculated from their free energies^[^ ^15^ ^]^

(1) $$\begin{array}{ccc} & & O_{2}+2H_{2}O+2e^{-}\to H_{2}O_{2}+2{\mathrm{OH}}^{-}\\ & & \;U^{0}=+0.70\;V\;\text{versus}\;\text{RHE}\;\left(\text{pH}≦11.6\right) \end{array}$$

(2) $$\begin{array}{ccc} & & O_{2}+H_{2}O+2e^{-}\to H{O_{2}}^{-}+OH^{-}\\ & & \;U^{0}=+0.76\;V\;\mathrm{versus}\;\mathrm{RHE}\;\left(\mathrm{pH}>11.6\right) \end{array}$$

In this study, H_2_O_2_ is regarded as equivalent to HO_2_^−^. For example, HO_2_^−^ formed at pH 12 was quantified by colorimetric titration after neutralizing the sample solution. The H_2_O_2_ production rates in air and under illumination were quantified, and examples are shown in Figure 2d. Surprisingly, the production rates achieved >40 000 mg (H_2_O_2_) g_photocat_^−1^ h^−1^ with a Faradaic efficiency of 96 ± 3% (at pH 12; see Table S1 in the Supporting Information). Long‐term durability testing under illumination at pH 12 and 0 V versus Ag/AgCl showed that PPT was robust for at least one week, with a turnover number exceeding 10^5^ and high recyclability (data for five runs are seen in Table S2 in the Supporting Information). The corresponding Raman spectra shown in Figure S3 (Supporting Information) did not show any changes in the chemical composition of PPT over the week‐long test, supporting its remarkable robustness. Figure 2a (light trace) suggests that this limited solubility of oxygen is indeed limiting the conversion current to remain below ≈75 µA cm^−2^. This means that, for the thicker films in Figure 2d, the H_2_O_2_ concentration exceeds the oxygen concentration after ≈15–20 min and thus makes the conditions more thermodynamically difficult than suggested by the *U*_0_ values at standard conditions.

PPT also acts as an electrocatalyst for oxygen reduction to H_2_O_2_ or HO_2_^−^ in the dark. In situ Raman spectroscopy of PPT suggested charge transfer via the phenylene groups of PPT as the catalytic sites after the application of a potential, indicating a two‐electron reduction per the repeating unit in the presence of oxygen (see Figures S4 in the Supporting Information). That is, light illumination solely results in the input of energy (voltage) to the system, and not the creation of new catalytic sites. While thiophenes have often been reported as the catalytic active sites for H_2_O_2_ production,^[^ ^12a,c^ ^]^ this experimentally proposed mechanism suggests that the benzene moiety is a more favorable site for catalytic H_2_O_2_ production than thiophene, presumably due to the chemical stability of its intermediate based on the resonance structure. The high stability of the intermediate may support the high catalytic ability of PPT at high pH. This implies the potential of the molecular design of more efficient and robust aromatic polymers as photocatalysts. The charge transfer via catalytic sites on the phenylene groups follows a completely different catalytic route from the light‐facilitated water reduction to H_2_ (on thiophene groups) previously reported on *π*‐conjugated polymers^[^ ^11f^ ^]^. (For the solution containing oxygen, the high catalytic activity for oxygen reduction to H_2_O_2_ prevents water reduction, as oxygen reduction proceeds at much more favorable potential; this is also supported by the very high Faradaic efficiency.)

The high oxygen reduction activity and chemical robustness of the PPT polymer under illumination at high pH allowed for its combination with traditional water‐oxidation catalysts to realize photochemical H_2_O_2_ production at high pH.

### 2.2 H_2_O_2_ Production in a Full‐Cell Setup with Zero Bias

**Figure 3:**
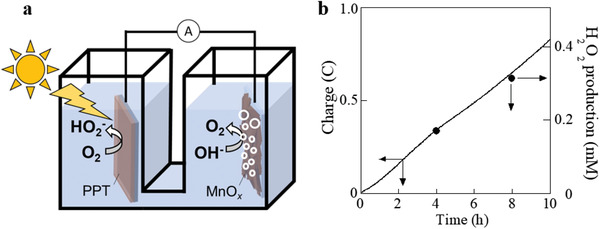
Measurement conducted using a two‐electrode photo‐electrochemical cell in the absence of a bias potential at pH 12 with PPT (under illumination) and MnO*_x_* as the cathode and anode, respectively. a) Scheme of the photo‐electrochemical measurement setup. b) Accumulated charge over time (solid line) based on CA measurements upon illumination at zero bias versus MnO*_x_* (pH 12) and H_2_O_2_ concentrations (points) as quantified by titration.

The unique properties of the PPT polymer layer make it suitable for combination with traditional high‐pH water‐oxidation catalysts. A two‐electrode photo‐electrochemical cell setup was first employed to explore the possibility of light‐driven H_2_O_2_ production at zero applied bias. Electrodeposited MnO*_x_* on a fluorine‐doped tin oxide (FTO) plate was used as a water‐oxidation catalyst^[^ ^9^ ^]^ and as both the counter and reference electrodes (**Figure** **3**a). A separate experiment surprisingly revealed that the electrodeposited MnO*_x_* electrode did not oxidize H_2_O_2_ under the alkaline conditions used (at least with less than 100 × 10^−3^ m H_2_O_2_; Figure S5, Supporting Information); thus, water oxidation on MnO*_x_* was assumed to be the only oxidative reaction occurring. (Iron oxides are known to decompose H_2_O_2_;^[^ ^16^ ^]^ therefore, we carefully selected MnO*_x_* as a water oxidation catalyst.) The time dependence of the accumulated charge from the 0 V CA measurements, i.e., zero‐volt bias (Figure 3b) clearly indicated current flow between the two half‐cells, thereby confirming the ability of PPT to produce a photovoltage sufficient for water oxidation on MnO*_x_*. Quantified H_2_O_2_ (or HO_2_^−^) production plots are also illustrated in Figure 3b. These closely resemble the amount of charge flow (or a Faradaic efficiency of 96 ± 2%), thereby confirming that oxidation of H_2_O_2_ on the MnO*_x_* anode must be practically absent. PPT achieved an outstanding H_2_O_2_ production rate of 11 000 mg (H_2_O_2_) g_photocat_^−1^ h^−1^ (chemically quantified) even without a bias voltage.

A recent study reported a photo‐electrocatalytic cell setup for H_2_O_2_ production by combining a photoanode and a cathode.^[^ ^17^ ^]^ In most cases, such combinations require the use of different electrolytes for the anode and cathode compartments, as well as metal complexes (not organic materials, as in this study) for the key reaction of oxygen reduction. The use of PPT as a photocatalytic cathode with a sufficient photovoltage to drive both the anode and cathode reactions eliminates the need for different electrolytes and a membrane separator between the anode and cathode compartments. A similar approach to photo‐electrochemical H_2_O_2_ production employed a combination of phthalocyanine, perylene carboxylic imide, and gold as a complex and highly unstable photocathode.^[^ ^18^ ^]^ In contrast, our cell uses a simple and robust polymer layer formed on a thin conducting plate as the photocathode and conventional MnO*_x_* electrodeposited on another plate as the anode, thus realizing photo‐electrochemical H_2_O_2_ production using only earth‐abundant elements. Although this simple two‐electrode setup is advantageous as a measuring system, it is limited in terms of the resistance of the electrolyte and the external circuit. To significantly reduce the related energy loss, a simpler and more direct combination of PPT and MnO*_x_* was subsequently implemented.

### 2.3 Complete Photocatalytic H_2_O_2_ Production on a Single Plate

To combine PPT and MnO*_x_* on the same electrically conductive thin plate (Figure 1b), MnO*_x_* was electrodeposited on one face of the plate, and a PPT layer was then formed on the opposite face via iodine vapor–assisted polymerization of dithienylbenzene (Figure S6, Supporting Information). The performance of the PPT photocatalyst combined with MnO*_x_* at pH 12 was investigated under 1.0 Sun irradiation (Figure 3a), and an outstanding H_2_O_2_ production rate of 14 000 mg (H_2_O_2_) g_photocat_^−1^ h^−1^ (230 mg (H_2_O_2_) g_photocat_^−1^ h^−1^ based on the combined weight of the anode and photocatalytic cathode catalysts; significant excess of MnO*_x_* vs the amount estimated for its single activity) or 0.20 mg (H_2_O_2_) cm^−2^(plate) h^−1^ was achieved and maintained for at least one week.

Figure 1a shows the reactions occurring over the PPT photocatalyst. The water oxidation potential at high pH was more negative than the HOMO level of PPT, which allowed for water oxidation on MnO*_x_*. At a lower pH, this was not the case, and photocatalytic H_2_O_2_ production was therefore impossible. The LUMO level of PPT is more than 1 V higher than the theoretical potential required for the conversion of O_2_ to H_2_O_2_ at high pH. This leaves ample room for the design of the copolymers of phenylene and thiophene with lower‐lying LUMOs, and hence lower bandgaps, to harvest longer wavelengths from the solar spectrum.

## 3 Conclusion

We demonstrated that the thiophene‐containing *π*‐conjugated organic polymer PPT works both as a visible‐light harvester and a catalyst for oxygen reduction to H_2_O_2_ or HO_2_^−^, so that the highest photo‐electrocatalytic conversion rate of >40 000 mg (H_2_O_2_) g_photocat_^−1^ h^−1^ could be achieved at 0 V versus Ag/AgCl and pH 12 with very high selectivity (>95%). PPT can be used in combination with a common and inexpensive water oxidation catalyst to produce H_2_O_2_ from oxygen and conventional alkaline water under visible light as the only energy input and air as a practical oxygen source. MnO*_x_* was used as the anode for water oxidation to yield additional oxygen. The photovoltage provided by PPT was sufficient to drive both oxygen reduction and water oxidation, thus resulting in a reaction for the overall system, i.e., only one excitation (“photon”) would be required—rather than two excitations as is, e.g., the case where both cathode and anode are photo‐electrodes and indeed in the photosynthesis. The produced H_2_O_2_ exists in the alkaline solution (in this typical study) in the form of HO_2_^−^, which is more stable ^[^ ^19^ ^]^ than H_2_O_2_ at pH 7 (e.g., half‐lives of 1.2 months and 48 h for HO_2_^−^ at pH 12 and H_2_O_2_ at pH 7, respectively). HO_2_^−^ in an alkaline solution is easily converted to the highly reactive H_2_O_2_ by facile neutralization. This aspect is another advantage of the proposed system for practical on‐site H_2_O_2_ production. Currently, we are preparing transparent plates composed of an electrically conducting plastic substrate sandwiched with PPT and MnO*_x_* layers and are examining the production of more concentrated H_2_O_2_ by the illumination of five‐layer stacked plates soaked in alkaline water.

The plate combining the PPT photocatalyst and MnO*_x_* is easy to fabricate, and requires no separators or membranes in the water pool, making it highly suitable for practical use. With this setup, a steady H_2_O_2_ production rate of 14 000 mg (H_2_O_2_) g_photocat_^−1^ h^−1^ can be achieved. Thus, the developed photocatalyst provides a simple, sustainable, and safe H_2_O_2_ production method to meet the rapidly growing demand for decentralized H_2_O_2_ production and use.

## 4 Experimental Section

##### Iodine Vapor–Assisted Polymerization to form PPT Layer

1,4‐Di‐(2‐thienyl)benzene was prepared via the Suzuki–Miyaura coupling of 1,4‐dibromobenzene and 2‐(4,4,5,5‐tetramethyl‐1,3,2‐diocaborolan‐2‐yl)thiophene, as described in the previous paper.^[^ ^11d^ ^]^ A chlorobenzene solution of dithienylbenzene was spin‐coated onto a glassy carbon, ITO, or FTO plate, which was then placed in a pre‐heated chamber with iodine at 90 °C for 1 h. The sample was washed and soaked in acetonitrile repeatedly, dried in a vacuum chamber at 90 °C, and thereafter stored in water. To prepare PPT and MnO*_x_* on the same plate (see Figure S6 in the Supporting Information), first, one face of the plate was masked with a Kapton film, and MnO*_x_* was electrodeposited on the other face (the preparation method of the MnO*_x_* layer is also described in the Supporting Information). After removal of the mask, the PPT layer (such as 149 nm) was formed on the opposite face via the iodine vapor–assisted polymerization of dithienylbenzene.

##### Photo‐Electrochemical Testing

Illumination was provided by an Asahi Spectra MAX‐302 300‐W Xe lamp with an equivalent power of 1.0 Sun at the distance from the PPT plate (5 cm). Glass was used as the electrochemical cell window to filter out deep UV light at *λ* < 320 nm. All the measurements were performed with reproducibility. The turnover number was calculated as the molar number of produced H_2_O_2_ divided by the molar number of the dithienylbenzene unit of PPT. The weight of the PPT layer specimen was determined by the layer area (2.5 cm^2^), average thickness, and density of a PPT fragment.

##### H_2_O_2_ Quantification

The produced amount of H_2_O_2_ was determined by a spectrophotometric method after adjusting the pH to 9 using the copper(I) complex of 2,9‐dimethyl‐1,10‐phenanthroline as the indicator.^[^ ^20^ ^]^ The calibration curve for dilute H_2_O_2_ was prepared using fresh and standard H_2_O_2_ solutions (3 wt% without any additives; see Figure S7 in the Supporting Information), and the pH was adjusted to 9 by an NaOH aqueous solution. The analytical errors were within ± 2%. The entire H_2_O_2_ quantification procedure was performed within 1 h.

##### H_2_O_2_ Statistical Analysis

Several PPT layers were prepared on a substrate, and the electrochemical tests were performed on the same PPT specimen, but a new area of the layer was employed for each (see the Supplementary Methods section in the Supporting information). The analytical errors were within ±2% in the H_2_O_2_ quantification (Figure S7, Supporting Information). The photochemical H_2_O_2_ production was examined at five cycles (Table S2, Supporting Information).

## Author Contributions

K.O. and B.W.‐J. contributed to experiment planning, experiment execution, data analysis, and manuscript preparation. H.N. contributed to experiment planning and manuscript preparation. All authors have reviewed and commented on the manuscript before publication.

## Conflict of Interest

The authors declare no conflict of interest.

## Supporting information

Supporting Information
