# Supplementary material for: Copolymer of Phenylene and Thiophene toward a Visible‐Light‐Driven Photocatalytic Oxygen Reduction to Hydrogen Peroxide
Source: Adv Sci (Weinh). 2021 Jan 20;8(5):2003077. doi: 10.1002/advs.202003077 (PMC7927612; doi:10.1002/advs.202003077)
Supplement: Supplementary file 1 — Supporting Information [file ADVS-8-2003077-s001.pdf]

© 2020 Wiley-VCH GmbH

ADVANCED SCIENCE
Open Access

Supporting Information

for *Adv. Sci.*, DOI: 10.1002/advs.202003077

Copolymer of Phenylene and Thiophene toward a Visible-Light-Driven Photocatalytic Oxygen Reduction to Hydrogen Peroxide

Kouki Oka, Hiroyuki Nishide, and Bjorn Winther-Jensen**

WILEY-VCH

© 2020 Wiley-VCH GmbH

Supporting Information

Copolymer of Phenylene and Thiophene toward a Visible-Light-Driven Photocatalytic Oxygen Reduction to Hydrogen Peroxide

Kouki Oka, Hiroyuki Nishide, and Bjorn Winther-Jensen**

Supplementary methods

Substrates

Each substrate was sonicated in chloroform for 1 h and rinsed with acetone prior to PPT and MnO_x formation. Glassy carbon, fluorine-doped tin oxide (FTO)-coated glass, and indium tin oxide (ITO)-coated glass were purchased from Alfa Aesar (Production code: 38023-GH), Nippon Sheet Glass (Production code: FTN 1.6), and Sigma Aldrich (Production code: 576352), respectively.

Electrochemical testing

Ag/AgCl and a Ti mesh or MnO_x were used as a reference and counter electrode, respectively. Measurements at different pHs were acquired using the same sample. Aqueous solutions with different pH levels were prepared by adding aqueous NaOH or H_2SO_4 to 0.01 M aqueous NaCl to maintain a moderate ionic strength for electrolysis. The pH of each electrolyte was tested with a Mettler Toledo pH meter. Tests were performed at various pHs on the same substrate with a PPT layer, where a new area was employed for each pH electrolyte. Air (oxygen/nitrogen gas mixture) was bubbled through the cell for 30 min prior to testing.

Preparation of MnO_x layers

The MnO_x layers were prepared via electrodeposition as described elsewhere.[1] Deliquesced salt of ethylammonium nitrate was first prepared by neutralising ethylamine with dilute nitric acid, followed by the removal of water by rotary evaporation under reduced pressure at 70 °C for 2 h. The deposition electrolyte was prepared by acidifying a solution of $Mn(CH_3COO)_2$ (10 mM, 50 mL) in water:ethylammonium nitrate (1:9, v/v) with HNO_3 (4.0 M, 125 μL). FTO glass with an area of 5 cm × 3 cm was used as the deposition substrate (deposition area = 3.0 cm × 3.0 cm). The MnO_x layers were electrodeposited in a three-electrode electrochemical setup at 120 °C by applying a constant current density of 200 μA cm^{-2} for 5 min, then were thoroughly rinsed with distilled water. Characterisation details of MnO_x can be found in our previous paper.[2]

Material characterisation

MALDI (Autoflex, Bruker) was performed on the PPT layer formed on an ITO substrate. X-ray photoelectron spectroscopy (XPS, JPS-9010TR) was performed with Mg K and Al K radiation. The XPS electron binding energies were referenced to the C 1s peak at 284.6 eV. The UV-vis absorption spectra of the PPT layers formed on glass slides were recorded using a JASCO V670 spectrophotometer. X-ray diffraction (XRD) was performed with a Rigaku RINT-Ultima diffractometer.

Scanning electron microscopy images (accelerating voltage = 5 kV) and energy-dispersive X-ray spectra (accelerating voltage = 25 kV) were obtained using Hitachi S-4500S and Hitachi S-3000N instruments, respectively. Renishaw inVia Reflex Raman systems with 785- and 532-nm diode lasers were used for Raman and in situ Raman characterisation, respectively, with the detailed procedures described in our previous papers.[2-3] The polymer layer thickness was measured using a KLA-Tencor instrument.

The MALDI spectrum of PPT gave peaks at 721.2–3365.6 (main peak at 1442.4, monomer segment peak at 240.4), indicating the formation of PPT with a high degree of polymerisation. XPS measurements gave only peaks assignable to C and S, with no peaks ascribable to contaminating metal species (such as Sn, Fe, or Pd) or residual oxidant (i.e. iodine) in the PPT layers (below the detection limit or no additional intensities after background subtraction). The HOMO and LUMO levels of PPT were calculated from its oxidation potential (+0.79 V vs. Ag/AgCl in a 0.1 M tetrabutylammonium perchlorate acetonitrile solution at a scan rate of 50 mV s^{-1}) and maximum absorption edge wavelength (570 nm). The XRD pattern of 1,4-di(2-thienyl)benzene gave d_{001} ~7° (2θ), d_{100} ~15° (2θ), and d_{101} ~23° (2θ), suggesting lamellar and fishbone packing among the neighbouring dithienylbenzene molecules. After the formation of PPT, all of the long-range ordering signals from the crystal structure disappeared in the XRD patterns, as has been described in our previous paper.[4]

Materials

1,4-Dibromobenzene, 2-(4,4,5,5-tetramethyl-1,3,2-diocaborolan-2-yl)thiophene, iodine, chlorobenzene, and ethylamine were purchased from Tokyo Chemical Industry. Nitric acid, $Mn(CH_3COO)_2$, NaOH, and H_2SO_4, were purchased from FUJIFILM Wako Pure Chemical Corporation. Standard H_2O_2 solutions were purchased from Millipore and Sigma-Aldrich.

Supplementary Figures

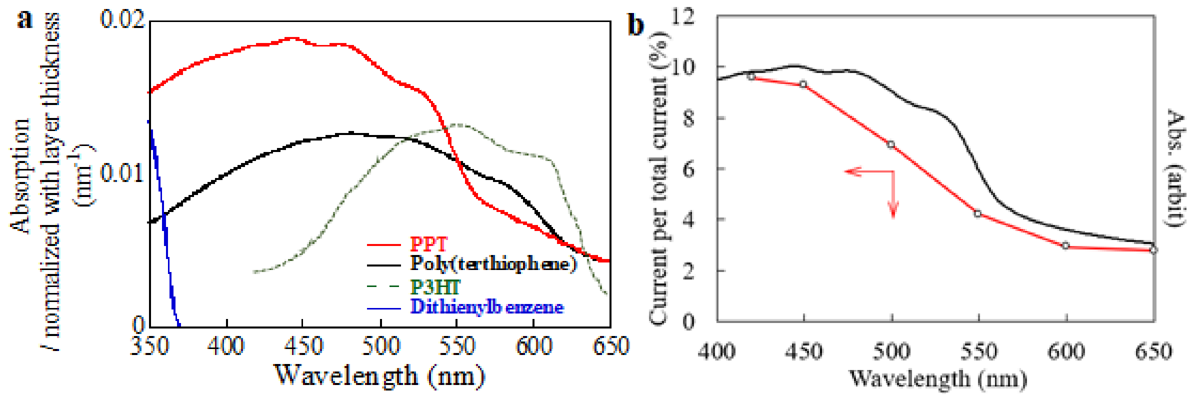

Figure S1. a**,** Normalized UV-Vis absorption spectra of a PPT layer (22 nm thickness), poly(terthiophene) layer (29 nm), and poly(3-hexylthiophene) (P3HT) layer (65 nm) cited from a previous paper[4]. b**,** Action spectrum of the PPT layer. The UV-vis spectrum of PPT exhibits additional peaks at a relatively high wavelength (520 nm), which suggests an additional π-orbital overlap.[5] No absorption in the range of 600–900 nm supported its un-doped state. PPT gave a higher absorbance than those of other typical polythiophenes. The UV-vis spectrum (arbitrary absorption) of dithienylbenzene (10 mg/mL) shows a single absorption peak at 340 nm.

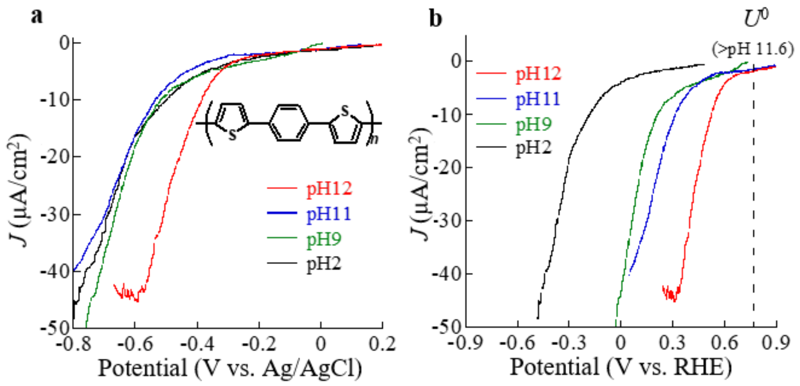

Figure S2. a, b, Linear sweep voltammograms recorded in the dark at 1 mV s^{-1} and different pHs for PPT as a cathode. *J* is current density calculated from the O_2 reduction reaction.

Electrocatalytic properties of PPT in the dark for H_2O_2 production

The ability of PPT to electrocatalytically reduce O_2 to H_2O_2 was also investigated in the dark. PPT layers formed on glassy carbon were electrochemically tested at pH 2–12. The electrochemical response at pH 12 was clearly different from those at lower pH, which was ascribed to the occurrence of different O_2 reduction reactions (Figure S2). At pH 12, O_2 reduction to HO_2^- is dominant, which leads to a different electrochemical behaviour. The overpotential of the PPT electrocatalyst decreased with increasing pH and approached zero at pH 12.

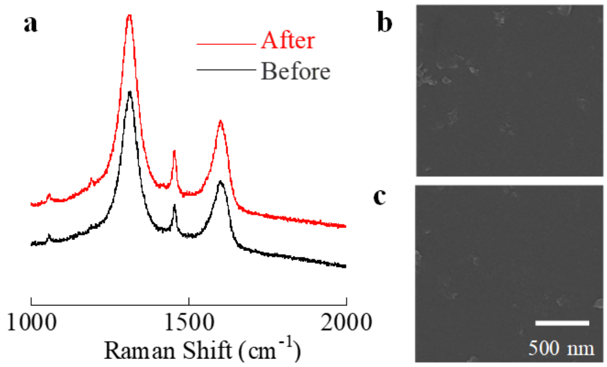

Figure S3. a, Raman spectra and SEM images of PPT b, before and c, after the long-term photoelectrochemical experiment at pH 12. The Raman spectra were measured with a 785-nm laser with no baseline correction. After the photoelectrochemical experiment was performed for one week, the concentration of H_2O_2 reached 2–10 mM at a stable H_2O_2 production rate, which is the same or greater than those (1–3 mM) of other state-of-the-art photocathodes with so-called high stabilities.[6] The Raman peaks at 1222, 1456, and 1600 cm^{-1} are assigned to C_α–C_α' ring stretching, C_α=C_β ring stretching, and C=C stretching (phenyl), respectively. The Raman spectra of PPT before and after the one-week photoelectrochemical experiment were identical, which indicated the robustness of the PPT chemical structure. The SEM image of PPT after the one-week photoelectrochemical experiment showed a well-maintained, uniform polymer layer with no delamination.

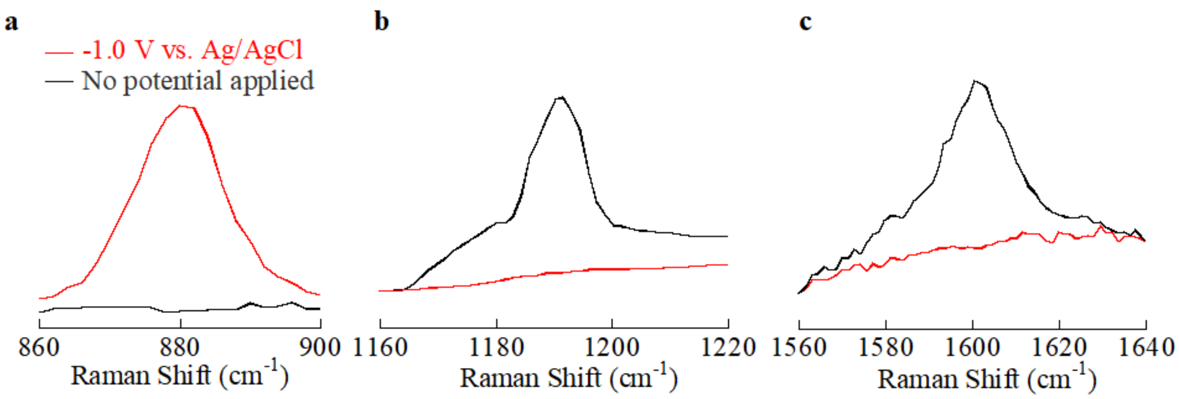

Figure S4. In situ Raman spectra of PPT at the range of a, 1160–1220 cm^{-1}, b, 1160–1220 cm^{-1} and c, 1560–1640 cm^{-1}. In situ Raman spectroscopy was performed according to our previous work[10]. The potentiostat and Raman spectrometer were carefully connected to eliminate any noise. The aqueous electrolyte was spread over the PPT layer, taking care to minimise any entry into the optical path. The in situ Raman spectra of PPT were acquired at pH 12 with a 532-nm laser. In situ Raman spectroscopy was applied to identify possible reaction sites and reaction intermediates and/or elucidate the chemical bonds formed/broken during the catalytic process. Analysis of the spectra recorded at −1.0 V vs. Ag/AgCl and pH 12 showed that the thiophene units remained unchanged during electrochemical H_2O_2 production, whereas the extent of the phenylene group conjugation decreased. The $C_\alpha(C_{\alpha'})$–C (phenylene) ring stretch at 1191 cm^{-1} disappeared and the OO stretch at 881 cm^{-1} appeared, while the phenylene C=C stretch at 1600 cm^{-1} decreased in intensity, which suggests that the PPT phenylene ring rather than the thiophene moiety was involved in the catalytic reduction of O_2 to H_2O_2.

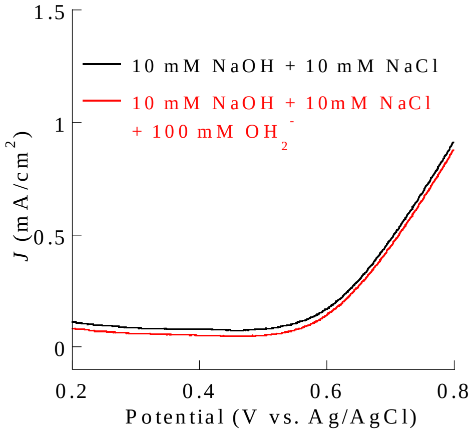

Figure S5. Linear sweep voltammograms of the MnO_x layer on a glassy carbon substrate in 10 mM NaOH and 10 mM NaCl aqueous electrolytes. Reference experiment with further addition of 100 mM H_2O_2 (in the form of O_2H^-) in the same solution. The scan rate was 10 mV s^{-1}.

CA of MnO_x at 0.7 V vs. Ag/AgCl and pH 12 using an electrochemical cell with a volume of 10 mL also showed oxygen evolution with a high Coulombic efficiency of 94%. Oxygen was detected by gas chromatography. Argon gas was bubbled through the cell for 30 min prior to oxygen detection to remove any oxygen in the cell from air. For further studies using H_2O_2 at higher concentrations (>1 M), it may be recommended to sandwich a separator between the working electrode and counter electrode to maintain a high Coulombic efficiency.

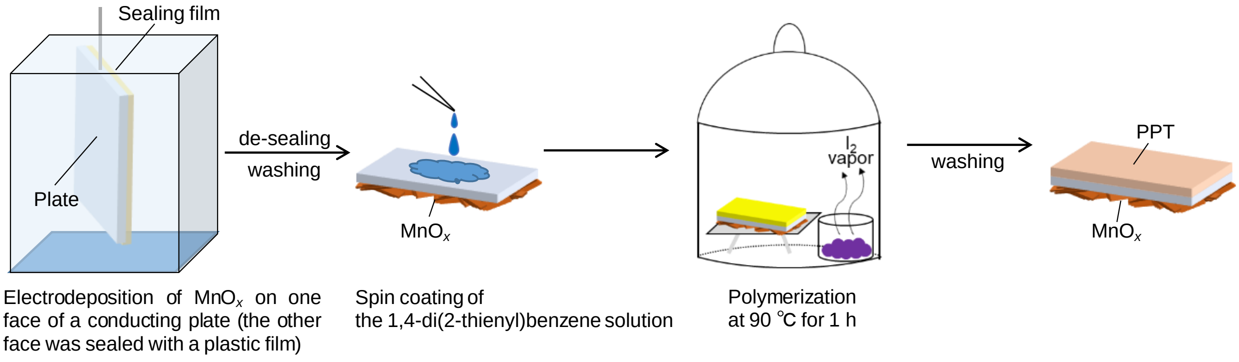

Figure S6. Schematic image of the preparation of PPT and MnO$_x$ on the same plate. One face of the plate was masked with a Kapton film, and MnO$_x$ was electrodeposited on the other face. After removal of the mask, a PPT layer was formed on the face opposite MnO$_x$ via the iodine vapor–assisted polymerization of dithienylbenzene.

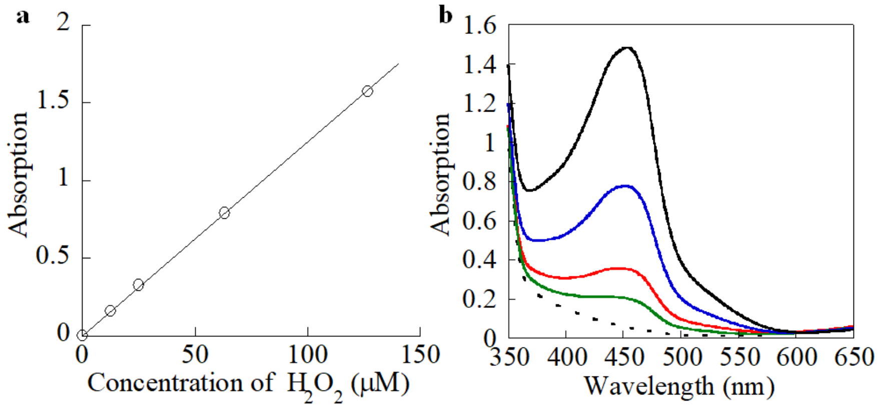

Figure S7. Calibration plots for the determination of H_2O_2 concentration. Dilute H_2O_2 was prepared using fresh and standard H_2O_2 solutions (3 wt% without any additives, Millipore), and the pH was adjusted to 9 by adding aqueous NaOH. The validity of the plots was checked with another standard H_2O_2 solution (3 wt%, Sigma Aldrich), and the error was within ± 2%. This spectrophotometric titration method to chemically quantify highly dilute H_2O_2 concentrations[7] is based on the simple, selective, and quantitative colour-changing reaction of the copper(I) complex of 2,9-dimethyl-1,10-phenanthroline with H_2O_2.

Supplementary Tables

Table S1. Photoelectrochemical H_2O_2 production

pH	Potential V vs Ag/AgCl	Overpotential V vs U^0	Gravimetric rate mg (H_2O_2) $g_{photocat}^{-1}$ h^{-1}
2	0	0.38	31,000
9	0	-0.04	39,000
12	0	-0.16	42,000
12	0.31	-0.47	11,000

Solutions with different pHs were prepared by adding aqueous NaOH or H_2SO_4 to 0.01 M aqueous NaCl to maintain a moderate ionic strength. The pH of each electrolyte was tested with a Mettler Toledo pH meter. A PPT layer with a thickness of 9.5 nm was used in these measurements. The experiments were performed at various pHs on the same PPT layer, with a new area employed for each electrolyte. Air (oxygen/nitrogen gas mixture) was bubbled through the cell for 30 min prior to testing. Illumination was provided by an Asahi Spectra MAX-302 300-W Xe lamp with an equivalent power of 1.0 sun at the distance of the PPT photocathode (5 cm). The glass electrochemical cell window filtered out deep UV light at $\lambda <$ 320 nm.

Table S2. Photoelectrochemical H_2O_2 production in the several cycles. At 0 V vs. Ag/AgCl and pH 12 using an electrochemical cell with a volume of 10 mL. A PPT layer with a thickness of 149 nm was used in these measurements. The experiments were performed at different cycles on the same PPT layer, with the same area employed for each electrolyte. Air (oxygen/nitrogen gas mixture) was bubbled through the cell for 30 min prior to testing. Illumination was provided by an Asahi Spectra MAX-302 300-W Xe lamp with an equivalent power of 1.0 sun at the distance of the PPT photocathode (5 cm). The glass electrochemical cell window filtered out deep UV light at λ < 320 nm.

Cycle	H_2O_2 Concentration after 10 h (mM)
1	0.34
2	0.35
3	0.35
4	0.34
5	0.34

References

[1] a) F. Zhou, C. McDonnell-Worth, H. Li, J. Li, L. Spiccia, D. R. Macfarlane, *Journal of Materials Chemistry A* **2015**, 3, 16642; b) F. Zhou, A. Izgorodin, R. K. Hocking, L. Spiccia, D. R. MacFarlane, *Advanced Energy Materials* **2012**, 2, 1013.

[2] K. Oka, O. Tsujimura, T. Suga, H. Nishide, B. Winther-Jensen, *Energy & Environmental Science* **2018**, 11, 1335.

[3] K. Oka, K. Noguchi, T. Suga, H. Nishide, B. Winther‐Jensen, *Advanced Energy Materials* **2019**, 9.

[4] K. Rahimi, I. Botiz, J. O. Agumba, S. Motamen, N. Stingelin, G. Reiter, *RSC Adv.* **2014**, 4, 11121.

[5] K. Oka, R. Kato, K. Oyaizu, H. Nishide, *Adv. Funct. Mater.* **2018**, 28.

[6] a) M. Jakešová, D. H. Apaydin, M. Sytnyk, K. Oppelt, W. Heiss, N. S. Sariciftci, E. D. Głowacki, *Adv. Funct. Mater.* **2016**, 26, 5248; b) O. Jung, M. L. Pegis, Z. Wang, G. Banerjee, C. T. Nemes, W. L. Hoffeditz, J. T. Hupp, C. A. Schmuttenmaer, G. W. Brudvig, J. M. Mayer, *J. Am. Chem. Soc.* **2018**, 140, 4079; c) M. Gryszel, A. Markov, M. Vagin, E. D. Głowacki, *Journal of Materials Chemistry A* **2018**, 6, 24709.

[7] A. N. Baga, G. R. A. Johnson, N. B. Nazhat, R. A. Saadalla-Nazhat, *Anal. Chim. Acta* **1988**, 204, 349.
